# Tyrosine Deprotonation and Associated Hydrogen Bond Rearrangements in a Photosynthetic Reaction Center

**DOI:** 10.1371/journal.pone.0026808

**Published:** 2011-10-24

**Authors:** Hiroshi Ishikita

**Affiliations:** 1 Career-Path Promotion Unit for Young Life Scientists, Graduate School of Medicine, Kyoto University, Kyoto, Japan; 2 Japan Science and Technology Agency (JST), PRESTO, Saitama, Japan; George Mason University, United States of America

## Abstract

Photosynthetic reaction centers from *Blastochloris viridis* possess Tyr-L162 located mid-way between the special pair chlorophyll (P) and the heme (heme3). While mutation of the tyrosine does not affect the kinetics of electron transfer from heme3 to P, recent time-resolved Laue diffraction studies reported displacement of Tyr-L162 in response to the formation of the photo-oxidized P^+•^, implying a possible tyrosine deprotonation event. p*K*_a_ values for Tyr-L162 were calculated using the corresponding crystal structures. Movement of deprotonated Tyr-L162 toward Thr-M185 was observed in P^+•^ formation. It was associated with rearrangement of the H-bond network that proceeds to P via Thr-M185 and His-L168.

## Introduction

**Figure 1:**
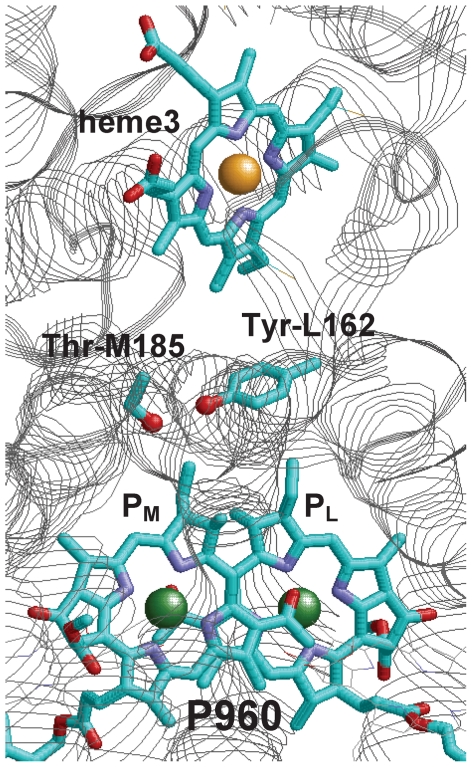
Special pair chlorophylls P960 and P680 and the electron donors heme3.

In biological systems, tyrosine residues often play an important role in functioning as a redox active group and mediating electron transfer. In photosystem II (PSII), electronic excitation of the chlorophyll *a* P680 P_D1/D2_ pair leads to formation of positively charged P680^+•^ as a consequence of electron transfer to the secondary quinone via the accessory chlorophyll *a*, a pheophytin *a*, and the primary quinone. The resulting P680^+•^ is reduced by D1-Tyr161 (Y_Z_) through electron transfer events from the Mn_4_CaO_5_ cluster [1]. The PSII reaction center that consists of D1 and D2 subunits has considerably large structural similarity with photosynthetic reaction centers from purple bacteria (bRC) [2]. In bRC from *Blastochloris viridis*, the corresponding chlorophyll pair is the bacteriochlorophyll *b* (BChl*b*) P_L/M_ pair P960 (P). The photo-oxidized P^+•^ state that is generated as a consequence of electronic excitation of P960 can be reduced by electron transfer from the nearest heme group (heme3) in the adjacent tetraheme subunit. The role of a highly conserved residue, Tyr-L162, has been long discussed [3], [4] due to its unique position halfway between heme3 and P (Figure 1). Nevertheless, in kinetic studies, the electron transfer rate from heme3 to P was not altered significantly in the Tyr-L162 mutations. Thus, it was concluded that neither tyrosine nor aromaticity is required for fast electron transfer from heme3 to P [5], [6]. Hence, functionally dominant electron transfer pathways may not proceed via Tyr-L162.

On the other hand, displacement of Tyr-L162 by 1.3 Å toward P^+•^ was very recently reported in the light-exposed crystal structure (light structure) with respect to the dark-state structure (dark structure) in time-resolved Laue diffraction analysis. Wöhri et al. interpreted that negatively charged and deprotonated Tyr-L162 was attracted to the P^+•^ positive charge [7]. Furthermore, they proposed that Tyr-L162 deprotonation may be important for the mechanism of electron transfer from heme3 to P via stabilization of heme3 in the oxidized state. A simple free energy calculation on the basis of molecular dynamics simulation is useful as an initial survey to roughly estimate the energetics of the tyrosine deprotonation. However, the energy profile is generally calculated in the fixed protonation pattern of the protein titratable residues. In particular, bRC possesses a number of titratable residues that can alter the protonation states in response to changes in redox states or protonation states of the cofactors or residues [8], [9], [10], [11], [12]. Apparently, the p*K*_a_ value of Tyr-L162 (p*K*_a_(Tyr-L162)) is neither experimentally measured nor explicitly calculated in Ref. [7], without considering the equilibrium in the strongly coupled protonation states of titeatable residues in the bRC protein environment.

Although there are crystal structures of bRC from *Blastochloris viridis* at higher resolutions, so far only the crystal structure by Wöhri et al. [7] was proposed to correspond to the photoactivated form. Notably, in their original structural studies [7], they discussed subtle differences in the orientation of the tyrosine side chain between the photoactivated form (PDB; 2X5V) and the dark form (PDB 2X5U), irrespective of the resolutions at ∼3 Å. Thus, it is a request from the community, at least once to evaluate i) p*K*_a_(Tyr-L162) in the original protein geometry of the photoactivated form and ii) what residues/groups contribute to downshift p*K*_a_(Tyr-L162). As a driving force of the tyrosine deprotonation, the P^+•^ state formation is definitely a key factor. However, there are also other titratable residues in the neighborhood of P. It is unclear whether protonation state changes of other titratable residues may occur in response to the P^+•^ formation, or whether deprotonation of other titratable residues compensate for the influence of P^+•^ on p*K*_a_(Tyr-L162).

To evaluate the energetics of Tyr-L162 deprotonation in the P^+•^ state formation, p*K*_a_(Tyr-L162) were calculated using the corresponding protein crystal structures, by solving the linear Poisson-Boltzmann equation with consideration of the protonation states of all titratable sites in the entire bRC protein. Using this approach, one will be able to sufficiently consider the equilibrium in protonation states of all titratable groups in bRC [9], [11] and clarify the factors (e.g., residues, cofactors, atomic charges, or hydrophobicity of the protein environment) that shift p*K*_a_(Tyr-L162) in the protein environment.

## Results and Discussion

### Movement of deprotonated tyrosine

**Figure 2:**
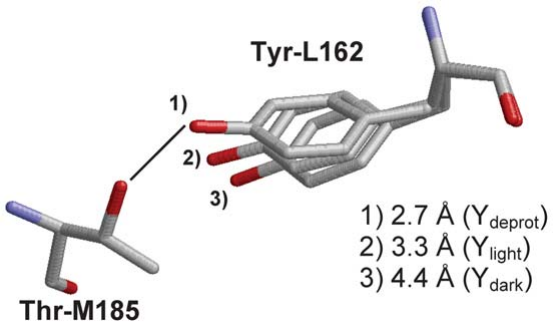
Variation of the Tyr-L162 side chain positions in the 1) Y_deprot_, 2) Y_light_, and 3) Y_dark_ conformers. The O_Thr-M185_–O_Tyr-L162_ distances are 2.5 Å (Y_deprot_), 3.3 Å (Y_light_), and 4.4 Å (Y_dark_). Note that the O_Ser-M188_–O_Tyr-L162_ distances are 5.3 Å (Y_deprot_), 5.7 Å (Y_light_), and 6.8 Å (Y_dark_).

To investigate the possible presence of deprotonated tyrosine, Tyr-L162 was treated in its deprotonated form, and its geometry was energetically optimized with CHARMM in the P^+•^ state. As a consequence, deprotonated Tyr-L162 moved further toward Thr-M185 (Figure 2): the H-bond distance between Tyr-L162 and Thr-M185 (O_Tyr-L162_-O_Thr-M185_) was 2.7 Å in the resulting geometry with deprotonated Tyr-L162 (Y_deprot_ position), which was 0.6 Å shorter than that in the light structure (Y_light_ position). Although the resulting Y_deprot_ position was not exactly identical to the Y_light_ position, this result implies that Tyr-L162 deprotonation leads to tyrosine movement from the one in the dark structure (Y_dark_ position) to the Y_light_ position. Interestingly, the O_Tyr-L162_-O_Thr-M185_ distance obtained with deprotonated Tyr-L162 is 2.7 Å (in the Y_deprot_ structure).

### Tyr-L162 deprotonation induces H-bond network rearrangements

**Figure 3:**
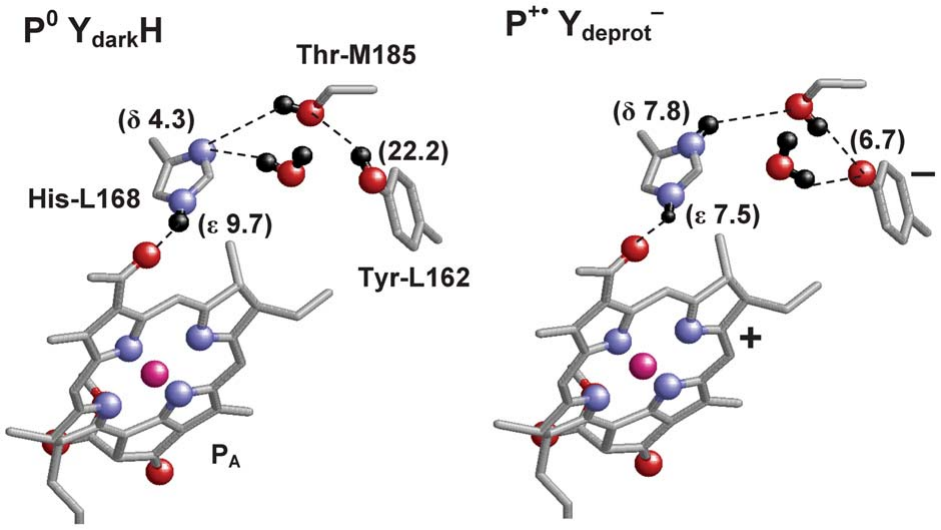
Hydrogen bonding pattern in the dark P^0^ Y_dark_H state (left) and the photooxidized P^+•^ Y_deprot_^−^ state (right). p*K*_a_ values are indicated in the bracket. Key hydrogen bonds are shown as dotted lines. For clarity, only one of the pair chlorophyll, P_A_ is shown in the figure.

In addition to Tyr-L162 movement, a striking rearrangement in the H-bond network containing P and Tyr-L162 was observed in the transition from the initial uncharged P^0^ and protonated Tyr-L162 state (P^0^ Y) to the photo-oxidized P^+•^ and deprotonated Tyr-L162 state (P^+•^ Y^−^). In the P^0^ Y_dark_ state (Figure 3, left), the hydroxyl H atom of Tyr-L162 can be oriented toward the hydroxyl O atom of Thr-M185 (O_Tyr-L162_-O_Thr-M185_ distance = 4.4 Å). The hydroxyl H atom of Thr-M185, in turn, is oriented to the Nδ site of His-L168 (O_Thr-M185_–N_His-L168_ distance = 4.3 Å), forming the O-H_Tyr-L162_ ••• O-H_Thr-M185_ ••• N_His-L168_ network over Tyr-L162, Thr-M185, and His-L168.

In contrast to the P^0^ Y_dark_ state, orientation of the H-bond network is completely different in the P^+•^ Y_deprot_^−^ state, since the hydroxyl OH group of Thr-M185 is subject to forming an H-bond with the deprotonated Tyr-L162 (O_Tyr-L162_-O_Thr-M185_ distance = 2.7 Å) to stabilize the negative charge (Figure 3, right). The absence of the hydroxyl H atom near His-L168 promotes protonation of the His-L168 Nδ site (Table 1). In accordance with reorientation of the Thr-M185 hydroxyl group, H atoms of a water molecule at an H-bonding distance with Tyr-L162 were also reoriented toward the deprotonated Tyr-L162. As a consequence, the OH dipole orientations were altered, forming the O_Tyr-L162_ ••• H-O_Thr-M185_ ••• H-N_His-L168_ network (Figure 3, right).

**Table 1:** Calculated p*K*_a_ (Tyr-L162, His-L168, and Glu-C254) and redox potential (Tyr-L162) values in mV and p*K*_a_ units, respectively.

		P^0^ Y_dark_H	P^+•^ Y_dark_^−^	P^+•^ Y_light_^−^	P^+•^ Y_deprot_^−^
Tyr-L162	p*K*_a_(YH/Y^−^)	22.2	13.7	10.8	6.7
His-L168	p*K*_a_(Nε)	9.7	6.2	6.3	7.5
	p*K*_a_(Nδ)	4.3	7.7	7.7	7.8

### p*K*_a_ (Tyr-L162) value shift from the P^0^ Y_dark_ to the P^+•^ Y_deprot_^−^ state

p*K*_a_ (Tyr-L162) was calculated to be 22 in the P^0^ Y_dark_ state (Table 1), indicating that this residue will never be deprotonated in the dark structure. The significantly high p*K*_a_(Tyr-L162) value of 22, which is even higher than that in aqueous solution (∼10), is mainly due to the presence of acidic residues in the bRC that upshift p*K*_a_(Tyr-L162), e.g., Asp-M182, Glu-C254, Asp-L155, and Glu-M171 (Table 2). The presence of these negatively charged acidic residues upshifts p*K*_a_ (Tyr-L162) and thus does not energetically allow deprotonated Tyr-L162 formation.

**Table 2:** Main residues that contribute to increase of p*K*_a_(Tyr-L162) in p*K*_a_ units (i.e., residues that stabilize the Tyr-L162 protonation state).

	P^0^ Y_dark_H			P^+•^ Y_deprot_^−^		
	side.^a^	b.b.^b^	total	side.^a^	b.b.^b^	total
Asp-M182	2.8	0.4	3.2	4.0	0.5	4.5
Glu-C254	2.3	−0.2	2.1	1.9	−0.1	1.8
Asp-L155	2.0	0.2	2.1	1.5	0.2	1.7
Asn-L158	1.3	0.4	1.7	1.1	0.5	1.6
Glu-M171	1.1	0.1	1.2	1.0	0.1	1.1

a Side chain.

b Backbone.

In contrast to the P^0^ Y_dark_ state, P^+•^ Y^−^ state formation leads to a drastic decrease in p*K*_a_ (Tyr-L162). In particular, the P^+•^ Y_deprot_^−^ state possesses the deprotonated Tyr-L162 since p*K*_a_ (Tyr-L162) = 6.7 (Table 1. Two major factors contribute to decreased p*K*_a_ (Tyr-L162):

#### i. H-bond pattern change

The most crucial groups that decrease p*K*_a_ (Tyr-L162) are Thr-M185 and a water molecule. They alter the H-bond pattern with respect to Tyr-L162 in response to the P^+•^ Y_deprot_^−^ state formation (Figure 3). As a consequence, H-bond alternation in Thr-M185 and a water molecule decrease p*K*_a_ (Tyr-L162) by 8 and 4 in the P^+•^ Y_deprot_^−^ state (relative to the P^0^ Y_dark_ state), respectively (Table 3).

**Table 3:** Main residues that contribute to decrease of p*K*_a_(Tyr-L162) in the P^+•^ Y^−^ state formation in p*K*_a_ units (i.e., residues that promote the Tyr-L162 deprotonation).

	P^0^ Y_dark_H			P^+•^ Y_dark_^−^			P^+•^ Y_light_^−^			P^+•^ Y_deprot_^−^		
	side.^a^	b.b.^b^	total	side.^a^	b.b.^b^	total	side.^a^	b.b.^b^	total	side.^a^	b.b.^b^	total
Thr-M185	0.9	0.0	0.9	−1.8	0.0	−1.8	−3.5	−0.4	−3.9	−6.4	−0.6	−7.1
His-L168	0.0	−0.4	−0.4	−0.3	−0.4	−0.7	−1.6	−0.5	−2.1	−3.9	−0.7	−4.6
Water-M2001			0.2			−1.5			−3.1			−3.8
P_A_			−0.1			−2.2			−2.5			−2.7
P_B_			0.1			−1.4			−1.6			−1.5
Arg-C264	−1.5	0.0	−1.6	−1.5	0.0	−1.6	2.3	−0.1	−0.1	−1.1	0.0	−1.1
Ser-M188	−0.9	−0.6	−1.4	−0.9	−0.6	−1.5	−0.8	−0.6	−1.6	−0.6	−0.5	−1.1
Arg-L135	−0.9	0.0	−1.0	−0.9	0.0	−1.0	−1.0	0.0	−1.0	−1.0	0.0	−1.0
Arg-M190	−1.0	−0.2	−1.2	−1.0	−0.2	−1.2	−0.9	−0.2	−1.1	−0.8	−0.2	−1.0

a Side chain.

b Backbone.

#### ii. Direct electrostatic influence of a positive charge in the photo-oxidized P^+•^ state

The positive charge on P^+•^ contributes to stabilization of the deprotonated Tyr-L162 form, downshifting p*K*_a_ (Tyr-L162) by 4.2 (2.7 from P_A_ and 1.5 from P_B_) in the P^+•^ Y_deprot_^−^ state (Table 3). The influence of P^+•^ on p*K*_a_(Tyr-L162) did not essentially differ in the Y_dark_, Y_light_, and Y_deprot_ positions (Table 3).

### Concluding Remarks

Deprotonation of Tyr-L162 resulted in the displacement of the side chain, lowering the p*K*_a_ value to 6.7. Movement of deprotonated Tyr-L162 toward Thr-M185 was observed in P^+•^ formation. It was associated with rearrangement of the H-bond network that proceeds to P via Thr-M185 and His-L168.

## Materials and Methods

### Atomic coordinates and charges

For performing computations of bRC from *Blastochloris viridis*, crystal structures in the photoactivated form (protein data bank (PDB); 2X5V) [7] were used. A crystal structure corresponding to the dark state is available (PDB 2X5U), but this crystal structure does not contain water molecules that can be seen in the photoactivated crystal structure. Furthermore, the conformer labeled with A in the photoactivated crystal structure is essentially identical to the dark state crystal structure in terms of the Tyr-L162 position while the conformer labeled with B in the photoactivated crystal structure is considered to correspond to the photoactivated state. Thus, in the present study, atomic coordinates for the A and B conformers (PDB 2X5V) were used as the dark and light structures, respectively.

The atomic coordinates were obtained using the same procedures used in previous studies (e.g., Refs. [11], [13], [14]). The positions of H atoms were energetically optimized with CHARMM [15] by using the CHARMM22 force field. While carrying out this procedure, the positions of all non-H atoms were fixed, and the standard charge states of all the titratable groups were maintained, i.e., basic and acidic groups were considered to be protonated and deprotonated, respectively. All of the other atoms whose coordinates were available in the crystal structure were not geometrically optimized. To investigate a possible movement of deprotonated Tyr-L162 (i.e., to yield the Y_deprot_ position, see the later part), atomic coordinates for the minimum set of relevant residues, i.e., Tyr-L162, Thr-M185, and a water molecule (HOH M 2001 in PDB: 2X5V) were released and geometrically optimized (Table S1 for atomic coordinates). As a general and uniform strategy, other crystal waters are removed in our computations [16] because of the lack of experimental information for hydrogen atom positions. Cavities resulting after removal of crystal water are uniformly filled with solvent dielectric of *ε* = 80.

Atomic partial charges of the amino acids were adopted from the all-atom CHARMM22 [15] parameter set. The charges of protonated acidic oxygen atoms in Asp and Glu were both increased symmetrically by +0.5 unit charges to account implicitly for the presence of a proton. Similarly, instead of removing a proton in the deprotonated state, the charges of all protons of the basic groups of Arg and Lys were diminished symmetrically by a total unit charge. For residues whose protonation states are not available in the CHARMM22 parameter set, appropriate charges were computed [17]. The atomic charges for the redox-active tyrosine (Tyr-L162) were adopted from the previous applications [16], [18] (deprotonated with negative charge (Y^−^), and protonated with neutral charge (YH)). The atomic charges of BChl*b* and bacteriopheophytin *b* (BPheo*b*) were determined from the electronic wave functions obtained with the density functional (DFT) module (B3LYP) in Gaussian03 [19] with 6-31G** basis set by fitting the resulting electrostatic potential in the neighborhood of these molecules by the RESP procedure [20] (Tables S2 and S3). To represent the charge states of the light-induced oxidized special pair P^+•^, a unit positive charge was distributed with a ratio of P_A_^+•^/P_B_^+•^ = 2/1 derived from ENDOR studies [21] as done in the previous application [22].

### p*K*a and protonation pattern

The present computation is based on the electrostatic continuum model by solving the linear Poisson-Boltzmann (LPB) equation with the MEAD program [23]. To facilitate a direct comparison with previous computational results, identical computational conditions and parameters were used (e.g., Refs. [11], [13], [14]) such as atomic partial charges and dielectric constants. The redox states of all other cofactors (i.e. accessory BChl*b*, BPheo*b*, and quinones) were kept in their neutral charge state. Hemes in the cytochrome *c* subunit were kept in the reduced state. The ensemble of the protonation patterns was sampled by the Monte Carlo method with Karlsberg [24] (Rabenstein, B. *Karlsberg online manual*, http://agknapp.chemie.fu-berlin.de/karlsberg/). The dielectric constants were set to *ε_p_* = 4 inside the protein and *ε_w_* = 80 for water. All computations were performed at 300 K, pH 7.0, and an ionic strength of 100 mM. The LPB equation was solved using a 3-step grid-focusing procedure at the resolutions 2.5 Å, 1.0 Å, and 0.3 Å. The Monte Carlo sampling for a redox active group yielded the probabilities [A_ox_] and [A_red_] of the two redox states of the molecule A.

To obtain absolute p*K*_a_ values of a target site (e.g. p*K*_a_(Tyr-L162)), the electrostatic energy difference was calculated between the two protonation states, protonated and deprotonated, in a reference model system using a known experimentally measured p*K*_a_ value. The difference in the p*K*_a_ value of the protein relative to the reference system was added to the known reference p*K*_a_ value. Experimentally measured p*K*_a_ values employed as references are 12.0 for Arg, 4.0 for Asp, 9.5 for Cys, 4.4 for Glu, 10.4 for Lys, 9.6 for Tyr [25], and 7.0 and 6.6 for deprotonation/protonation at Nε and Nδ atoms of His, respectively [26], [27], [28]. All of the other titratable sites were fully equilibrated to the protonation state of the target site during the titration. The Monte Carlo sampling for a titratable residue yielded the probabilities [protonated] and [deprotonated] of the two protonation states of the molecule. The p*K*_a_ value was evaluated using the Henderson-Hasselbalch equation. A bias potential was applied to obtain an equal amount of both protonation states ([protonated] = [deprotonated]), yielding the p*K*_a_ value as the resulting bias potential.

### Error estimation

The procedures to compute p*K*_a_ of titratable residues are equivalent to those of the redox potential for redox-active groups, although in the latter case, the Nernst equation is applied instead of the Henderson-Hasselbalch equation [29]. Therefore, the accuracy of the present p*K*_a_ computations is directly comparable to that obtained for recent computations [16]. From the analogy, the numerical error of the p*K*_a_ computation can be estimated to be about 0.2 pH units. Systematic errors typically relate to specific conformations that may differ from the given crystal structures.

## Supporting Information

Table S1 Energetically minimized atomic coordinates of Tyr-L162 (Y_deprot_), Thr-M185, and a water molecule.
(DOC)

Table S2 Atomic partial charge of BChl*b*.
(DOC)

Table S3 Atomic partial charge of BPheo*b*.
(DOC)
