# Supplementary material for: Tyrosine Deprotonation and Associated Hydrogen Bond Rearrangements in a Photosynthetic Reaction Center
Source: PLoS One. 2011 Oct 24;6(10):e26808. doi: 10.1371/journal.pone.0026808 (PMC3200362; doi:10.1371/journal.pone.0026808)
Supplement: Table S1 — Energetically minimized atomic coordinates of Tyr-L162 (Ydeprot), Thr-M185, and a water molecule. (DOC) [file pone.0026808.s001.doc]

Table S1. Energetically minimized atomic coordinates of Tyr-L162 (Y_deprot_), Thr-M185, and a water molecule.

| **residue** | | |  | **x** | **y** | **z** |
| --- | --- | --- | --- | --- | --- | --- |
| TYR | L | 162 | N | 33.538 | 35.088 | 55.868 |
| TYR | L | 162 | CA | 33.941 | 35.805 | 57.07 |
| TYR | L | 162 | CB | 32.74 | 36.574 | 57.716 |
| TYR | L | 162 | CG | 32.482 | 37.859 | 56.943 |
| TYR | L | 162 | CD1 | 33.446 | 38.877 | 56.974 |
| TYR | L | 162 | CD2 | 31.342 | 38.063 | 56.149 |
| TYR | L | 162 | CE1 | 33.368 | 39.987 | 56.115 |
| TYR | L | 162 | CE2 | 31.243 | 39.195 | 55.312 |
| TYR | L | 162 | CZ | 32.29 | 40.118 | 55.238 |
| TYR | L | 162 | OH | 32.283 | 41.142 | 54.259 |
| TYR | L | 162 | C | 34.71 | 34.907 | 58.051 |
| TYR | L | 162 | O | 35.142 | 35.342 | 59.12 |
|  |  |  |  |  |  |  |
| THR | M | 185 | N | 31.104 | 45.714 | 52.851 |
| THR | M | 185 | CA | 30.902 | 45.103 | 54.16 |
| THR | M | 185 | CB | 32.215 | 44.617 | 54.763 |
| THR | M | 185 | OG1 | 32.873 | 43.733 | 53.859 |
| THR | M | 185 | CG2 | 31.987 | 43.835 | 56.06 |
| THR | M | 185 | C | 30.153 | 46.029 | 55.119 |
| THR | M | 185 | O | 29.2 | 45.609 | 55.776 |
|  |  |  |  |  |  |  |
| HOH | M | 2001 | O | 34.288 | 40.177 | 52.852 |
