# Supplementary material for: Tyrosine Deprotonation and Associated Hydrogen Bond Rearrangements in a Photosynthetic Reaction Center
Source: PLoS One. 2011 Oct 24;6(10):e26808. doi: 10.1371/journal.pone.0026808 (PMC3200362; doi:10.1371/journal.pone.0026808)
Supplement: Table S3 — Atomic partial charge of BPheob. (DOC) [file pone.0026808.s003.doc]

Table S3. Atomic partial charge of BPheo*b*.

| **atom** | **BPheo*b*** | **BPheo*b*^–^** |  | **atom** | **BPheo*b*** | **BPheo*b*^–^** |
| --- | --- | --- | --- | --- | --- | --- |
| NA | -0.16 | -0.19 |  | CMA | -0.42 | -0.33 |
| NB | -0.11 | -0.10 |  | HMA1 | 0.10 | 0.07 |
| HNB | 0.17 | 0.18 |  | HMA2 | 0.10 | 0.07 |
| NC | -0.15 | -0.17 |  | HMA3 | 0.10 | 0.07 |
| ND | -0.02 | 0.03 |  | CBA | -0.55 | -0.51 |
| HND | 0.06 | 0.05 |  | HBA1 | 0.14 | 0.12 |
| C1A | -0.14 | -0.17 |  | HBA2 | 0.14 | 0.12 |
| CHA | 0.11 | 0.13 |  | CGA | 0.85 | 0.80 |
| C4D | 0.01 | -0.05 |  | O1A | -0.53 | -0.55 |
| C1B | 0.06 | 0.04 |  | O2A | -0.39 | -0.31 |
| CHB | -0.27 | -0.34 |  | C1 | 0.01 | -0.08 |
| HHB | 0.15 | 0.15 |  | H1 | 0.07 | 0.10 |
| C4A | 0.05 | 0.03 |  | H2 | 0.07 | 0.10 |
| C1C | -0.04 | -0.10 |  | C2B | 0.20 | 0.16 |
| CHC | -0.23 | -0.27 |  | CMB | -0.38 | -0.30 |
| HHC | 0.09 | 0.07 |  | HMB1 | 0.12 | 0.08 |
| C4B | 0.13 | 0.14 |  | HMB2 | 0.12 | 0.08 |
| C1D | 0.03 | -0.01 |  | HMB3 | 0.12 | 0.08 |
| CHD | -0.28 | -0.32 |  | C3B | -0.33 | -0.41 |
| HHD | 0.15 | 0.15 |  | CAB | 0.66 | 0.68 |
| C4C | 0.20 | 0.18 |  | OBB | -0.49 | -0.55 |
| C2A | 0.25 | 0.14 |  | CBB | -0.36 | -0.36 |
| H2A | 0.02 | 0.05 |  | HB1 | 0.10 | 0.08 |
| CAA | -0.01 | 0.00 |  | HB2 | 0.10 | 0.08 |
| HAA1 | 0.03 | 0.03 |  | HB3 | 0.10 | 0.08 |
| HAA2 | 0.03 | 0.03 |  | C2C | 0.44 | 0.57 |
| C3A | 0.19 | 0.25 |  | H2C | -0.01 | -0.05 |
| H3A | 0.01 | -0.02 |  | CMC | -0.39 | -0.39 |

| **atom** | **BPheo*b*** | **BPheo*b*^–^** |
| --- | --- | --- |
| HMC1 | 0.10 | 0.09 |
| HMC2 | 0.10 | 0.09 |
| HMC3 | 0.10 | 0.09 |
| C3C | -0.29 | -0.32 |
| CAC | -0.03 | -0.05 |
| HAC | 0.10 | 0.08 |
| CBC | -0.18 | -0.17 |
| HBC1 | 0.07 | 0.05 |
| HBC2 | 0.07 | 0.05 |
| HBC3 | 0.07 | 0.05 |
| C2D | 0.17 | 0.14 |
| CMD | -0.32 | -0.27 |
| HMD1 | 0.11 | 0.08 |
| HMD2 | 0.11 | 0.08 |
| HMD3 | 0.11 | 0.08 |
| C3D | -0.26 | -0.29 |
| CAD | 0.61 | 0.60 |
| OBD | -0.47 | -0.53 |
| CBD | -0.52 | -0.52 |
| HBD | 0.20 | 0.18 |
| CGD | 0.71 | 0.71 |
| O1D | -0.50 | -0.52 |
| O2D | -0.31 | -0.31 |
| CED | -0.01 | 0.02 |
| HED1 | 0.08 | 0.06 |
| HED2 | 0.08 | 0.06 |
| HED3 | 0.08 | 0.06 |
|  |  |  |
